# Effects of duration of untreated psychosis on long-term outcome of people hospitalized with first episode schizophrenia

**DOI:** 10.4103/0019-5545.64583

**Published:** 2010

**Authors:** Amresh Shrivastava, Nilesh Shah, Megan Johnston, Larry Stitt, Meghana Thakar, Gurusamy Chinnasamy

**Affiliations:** Department of Psychiatry, Schulich School of Medicine and Dentistry, The University of Western Ontario, London, Ontario, Canada; and Lawson Health Research Institute, London, Ontario, Canada; 1 LTMG Hospital, University of Mumbai, Sion, Mumbai-400 022, Maharashtra, India; 2 Department of Psychology, University of Toronto, Toronto, ON, Canada; 3 Department of Epidemiology and Biostatistics, Schulich School of Medicine and Dentistry, The University of Western Ontario, London, Ontario, N6A 5C1, Canada; 4 Mental Health Foundation of India (PRERANA Charitable Trust) and Silver Mind Hospital, 209 Shivkripa Complex, Gokhale Road, Thane, Mumbai-400 602, Maharashtra, India; 5 novaNAIT - Centre for Applied Research and Technology Transfer, The Northern Alberta Institute of Technology, 10504 Princess Elizabeth Avenue, Edmonton, Alberta, Canada T5G 3K4

**Keywords:** Duration of untreated psychosis, first-episode schizophrenia, long-term outcome

## Abstract

Duration of untreated psychosis (DUP) has emerged as a reliable predictor of outcome but continues to remain under scientific scrutiny. The present study examines the effect of differential periods of DUP on long-term outcome of first episode schizophrenia at Mumbai, India. This research was a prospective, 10-year follow-up naturalistic study. Hospitalized patients of first episode schizophrenia were selected and followed up. Results showed that the mean DUP was higher for a group which showed clinical recovery on Clinical Global Impression Scale [14.0 months (SD=8.0) in recovered and 10.8 months (SD=5.7) in non-recovered group (*P*=0.091)]. DUP was not found to be significantly associated with any of the end point parameters of good clinical or social outcome. Thus, this study found that DUP alone does not determine outcome status confirming the role of psychopathological heterogeneity.

## INTRODUCTION

Outcome of schizophrenia has been repeatedly demonstrated to be ‘good’ and ‘favorable,’ which generally implies that most of the patients treated adequately are able to maintain a reasonable quality of life, remain free from distressing symptoms, can function at a moderate level and live a life outside psychiatric institutions in the community.[1–5]

A number of reasons have been cited for this premise, which of course has currently come under some scrutiny.[6–7] There has been intense interest in duration of untreated psychosis (DUP) because of the proposal that psychosis is somehow neurologically toxic.[8] If this is true that delay in treating people with psychosis could impair prognosis, while reducing delay could improve it.[9] However, despite the blossoming of early intervention services, there is continuing disagreement over whether there is a real association between DUP and outcome. Several conflicting evidence have been reported.[10–12]

Although DUP has been reported as an independent marker of outcome, measurement errors and variability in DUP in terms of heterogeneity have also been reported and caution advised.[13,14] The strength of association between DUP and outcome has been found to be only ‘moderately strong’ based upon the available data, accounting for approximately 13% of variance or one-third to one-fourth of those who did not achieve remission.[15] Until now, very few long-term studies have examined this association. Long-term outcome of schizophrenia is multifactorial in nature; it not clearly known if a short DUP is a strong determinant of long-term outcome.[16] The present study examines the effects of DUP on clinical and social outcome in a 10-year, long-term follow-up in a cohort of first episode psychosis.

## MATERIALS AND METHODS

### Design

This study is a naturalistic, prospective, longitudinal follow-up study conducted at Mumbai, India. Assessments were conducted at the baseline and at the end of 10 years, follow-up, by trained and experienced clinical research staff. Inter-rater reliability was established for quantification of outcome.

### Sample and settings

Two hundred patients admitted with first episode psychosis were recruited as per inclusion criteria, and 101 were available at the end point. Wherever necessary patients were traced, contacted and assessed. The study was carried out in a non-governmental, psychiatric hospital certified as a psychiatric facility by the State Government as per Indian Mental Health Act 1983 from a period of 1993 to 2007. The Independent Ethics Commission of Mumbai approved the study.

All patients and their relatives were explained the nature and purpose of study and an informed consent was obtained at the beginning of the study as well as at the end of the follow up for repeat assessment.

### Inclusion and exclusion criteria

Baseline inclusion criteria included: hospitalization, availability of key relatives, confirmed diagnosis of psychotic disorder-non-affective as per Diagnostic and Statistical Manual (DSM-III-R) criteria; between the ages of 18-45 years, informed consent for participation in the study. Inclusion criteria at the end point of the study included: reconfirmed diagnosis of schizophrenia as per DSM IV -TR[17] at the follow-up of 10 tears; informed consent, and available objective data from key relatives.

We excluded cases of primary organic psychotic disorder intellectual disability, drug and substance induced psychosis, any change in diagnosis from baseline to endpoint, epilepsy, co morbid alcoholism and substance abuse.

### Assessment of DUP

The assessment of duration of untreated psychosis was done clinically by a detailed interview with the patient and the key relatives. We carefully assessed known prodromal signs and tried to elicit the time of first-distressing symptoms either positive or negative symptom to decide the onset of illness. The assessment of DUP included positive symptoms (hallucinations, delusions, and odd beliefs thought disorder), negative symptoms (depression, dysphoria, apathy, anergia, apathy, and amotivation), and social decline (withdrawn behavior, poor interpersonal relationship, social avoidance, and lack of interest in education or work).

### Assessment tools

We used clinical and social outcome criteria based upon Meltzer’s[18] criteria recommendations. We operationalized the definition on a scale of 1-to-5 where one represented poorest and 5 the best outcome for some of the parameters. This scale was developed for the local conditions and used in other studies.[19] Clinical outcome was measured by 1) Clinical Global Impression Scale (CGIS),[20,21] 2)Psychopathology (positive symptoms, negative symptoms and disorganization) using Positive and Negative Syndrome Scale [PANSS),[22,23] 3) Depressive symptoms using Hamilton Depression Rating Scale (HDRS)[24] 4) Factors of Compliance, 5) Extrapyramidal symptoms (EPS), using Abnormal Involuntary Movement Scale (AIMS)[25] 6) Aggression, 7) Hospitalization, and 8] Suicidality. Social outcome was measured using 1)Quality of life (QOL),[26] 2) Global Functioning (GAF),[27,28] 3)Independent living, 4) Family burden, and 5) Social burden by measured operationalized criteria. Raters in this study were not blinded.

### Outcome criteria

We used GCIS for measuring severity as well as improvement by CGIS-S and CGIS-I respectively. Primary criteria – a score of 2 or less i.e. scoring ‘improved and much improved’ rating were considered ‘good outcome’ on CGIS. Secondary outcome criteria included clinical improvement as defined by: 1) no hospitalization for minimum 2 preceding years, 2) GAF score less than 80, 3) QOL score greater than 80, 4) a score greater than 3 on scales of social functioning, independent living, education, and social burden.

## RESULTS

The statistical analysis was performed using SAS, version 9.1. Probability values less than 0.05 were considered to be statistically significant. Mean duration of untreated psychosis was observed as 12.7 months (SD =7.3). The majority of patients (73%) had duration of untreated psychosis ranging between six months to 24 months [Table 1]. There were no differences between short and long DUP in terms of age at intake and gender (Table 2, *P* =0.148 and *P* =0.799, respectively). No statistically significant differences were observed between the two groups on parameters of clinical and social recovery [Table 3].

**Table 1:** Duration of untreated psychoses on differential time line

Parameter	Value (SD)
Mean (SD)	12.7 (7.3)
Median (Minimum, Maximum)	11.0 (3, 35)
≤6 months	20 (19.8%)
6-11 months	34 (33.7%)
12-24 months	40 (39.6%)
>24 months	7 (6.9%)

**Table 2:** Differences in gender and age at intake between subjects with short and long dup(<12 months vs ≥ 12 months)

Outcome	<12 months DUP (n=54)	≥12 months DUP (n=47)	Test statistic	*P* value
Age at intake	27.7 (7.4)	30.1 (9.0)	t_98_=1.46	0.148
Male gender	39 (72.2%)	35 (74.5%)	X_1_^2^=0.06	0.799

**Table 3:** Difference in effect of duration of untreated psychoses on follow-up outcomes on multiple clinical and social parameters using 12 months cut-off for short and long DUP

Outcome	<12 months DUP (n=54)	≥12 months DUP (n=47)	Test statistic	*P* value
PANNS	52.4 (9.4)	50.6 (8.3)	t_99_=0.99	0.326
Positive symptoms	9.1 (4.1)	8.2 (3.7)	t_99_=1.17	0.244
Negative symptoms	12.8 (8.0)	11.5 (6.7)	t_99_=0.91	0.363
General				
Psychopathology	27.9 (11.5)	30.6 (12.2)	t_99_=1.11	0.270
HDRS	13.1 (5.2)	13.2 (5.3)	t_95_=0.18	0.861
GAF	77.6 (13.1)	80.5 (9.6)	t^94^=1.22	0.226
QOL	65.9 (14.1)	69.3 (15.2)	t_98_=1.16	0.248
Disorganization	25 (46.3%)	19 (40.4%)	X_12_=0.35	0.553
abnormal (>3)				
>1 Hospitalization	34 (64.2%)	27 (58.7%)	X_12_=0.31	0.578
in past 10 years				
IP Social abnormal	37 (68.5%)	36 (76.6%)	X_12_=0.82	0.366
(≤3)				
Work abnormal	44 (81.5%)	31 (67.4%)	X_12_=2.63	0.105
(≤3)				
EPS abnormal (>2)	18 (34.6%)	17 (36.2%)	X_12_=0.03	0.872
Independent living	26 (49.1%)	25 (54.4%)	X_12_=0.28	0.599
abnormal (≤3)				
Aggression	20 (37.0%)	19 (41.3%)	X_12_=0.19	0.663
abnormal (>2)				
Family burden	33 (63.5%)	21 (47.7%)	X_12_=2.40	0.122
abnormal (>3)				
Suicidality	28 (53.9%)	23 (52.3%)	X_12_=0.02	0.878
abnormal (2-5)				
Recovered	29 (53.7%)	32 (68.1%)	X_12_=2.17	0.141
(CGI -I <3)				

## DISCUSSION

There is a well-established association between DUP, critical period and early intervention. This association is independent of confounding factors, including premorbid functioning, gender, diagnosis and age of onset of symptoms variance in functional recovery has been reported.[29]

The finding of 48 weeks DUP in the present study is not surprising from a developing country where stigma is rampant, awareness is poor, accessibility of care is limited and resources for mental health are less than sufficient. A DUP as much as 796 weeks has been reported from India which is primarily because of lack of availability and accessibility of mental health services rather than the psychosis remaining ‘unidentified’.[30,31] Mental illness remains untreated despite recognition. There are several cultural, social, religious, economic and personal factors which determine approach to mental healthcare, which obliviously leads to longer DUP.[32] Long DUP has also been reported in western literature e.g. a Canadian study observed duration of untreated psychosis as 84 weeks.[33]

In the present study, in a multivariate analysis, results did not show any statistically significant correlation between various categories of duration of untreated psychosis and outcome parameters. The significant findings were the lack of correlation with symptom remission and level of social functions measured by several psychosocial parameters. We compared patients with less than 12 months of DUP and more than 12 months of DUP and found that no clinical or social parameters at ten years outcome correlated DUP below 12 months or more than 12 months. This lack of association may arise from the complexity inherent to the assessment of DUP or the fact that treatment may be inadequate due to limited resources. Additionally, the long-term outcome in schizophrenia is not influenced by DUP because most of neuronal changes take place early in the course or even preceding the onset and therefore an intervention as late as 12 months does not contribute to long term outcome.[34] DUP remains relevant only for short period of follow-up and once the psychosis has persisted long enough, enough toxic damage has been caused to change anything in the outcome.

The findings also indicate that longer the DUP worse the outcome but a shorter DUP does not necessarily mean a good outcome. Further, in our study out of 13 outcome parameters of clinical and social relevance none of the parameter showed any correlation. All the parameters most importantly, social function, global function, quality of life and independent living show no correlation. It is likely that DUP correlates with outcome measures in conjunction with several other factors. It further suggests that the benefit of early intervention in long term is gradually lost, no matter when the intervention is done due to several factors such as, poor treatment, lack of follow-up, inconsistencies in management, poor adherence, poor psychosocial intervention and frequent relapses. The assumption that delay in treating people with psychosis could impair psychosis while reducing delay would improve it, is not as straight forward as often stated.[35] There has been continuing disagreement over whether there is a real association between DUP and outcome.[12,36] We need more studies comparing ultra short DUP, short DUP and long DUP to understand more clearly about its association with outcome. Further studies also need to examine how powerful DUP is as a predictor.[37] Success of this concept depends upon public campaign and resources for treatments. Research of DUP has given a new responsibility for community awareness programs for early identification, which remains a daunting, task everywhere.[38,39]

## CONCLUSIONS

Our study finds that DUP alone does not determine long term outcome status in first episode schizophrenia. Long DUP leads to poor outcome and the short DUP does not necessarily lead to good outcome due to psychopathological heterogeneity in early phase.[30,31] There is a missing link in association of DUP and outcome.
